# Appearance and Speech Satisfaction and Their Associations With Psychosocial Difficulties Among Young People With Cleft Lip and/or Palate

**DOI:** 10.1177/1055665620926083

**Published:** 2020-05-28

**Authors:** Sarah N. Kelly, Joanna Shearer

**Affiliations:** 1 Department of Psychology, University of Bath, United Kingdom; 2 Department of Paediatric Psychology, Great Ormond Street Hospital, London, United Kingdom

**Keywords:** appearance satisfaction, speech satisfaction, psychosocial difficulties, cleft lip and palate

## Abstract

**Objective:**

Previous research has found lower appearance and speech satisfaction among those with cleft lip and/or palate (CL/P) compared to noncleft control groups. Less research has been conducted into which groups report the lowest satisfaction and how these two factors relate to psychosocial difficulties. This study aimed to investigate (1) differences in appearance and speech satisfaction by diagnosis and age among young people with CL/P and (2) associations between appearance and speech satisfaction and emotional and social difficulties.

**Design:**

Self-report questionnaires that had been collected between June 2016 and August 2018 within routine clinical practice were analyzed.

**Setting:**

A tertiary pediatric hospital in London.

**Patients:**

A total 130 nonsyndromic 10- and 15-year-old patients with CL/P.

**Main Outcome Measures:**

Cleft Hearing, Appearance and Speech Questionnaire; Strengths and Difficulties Questionnaire.

**Results:**

The CLP group reported significantly lower appearance satisfaction compared to the CP group (*P* = .005). The 15-year-olds reported significantly lower appearance satisfaction compared to the 10-year-olds (*P* = .008). No significant differences were found in speech satisfaction by diagnosis (*P* = .06) or age (*P* = .064). Significant negative associations were found at 15 years old between appearance satisfaction and emotional difficulties, speech satisfaction and emotional difficulties, appearance satisfaction and social difficulties, and speech satisfaction and social difficulties (*P* < .05 all correlations). Only the latter two associations were significant at 10 years old (*P* < .05).

**Conclusions:**

The findings have important implications as appearance and speech dissatisfaction may be ways in which to identify those at risk of psychosocial difficulties within clinical settings.

## Introduction

Cleft lip and/or palate (CL/P) is the most common congenital craniofacial anomaly, with overall prevalence estimated to be 1 in every 700 live births (World Health Organisation, 2001). A child with cleft can present with cleft lip, cleft palate, or cleft lip and palate. These congenital malformations can affect the way children look and talk, and it is assumed that this can have important consequences for psychosocial development.

Common characteristics of children with lip involvement include visible scarring around the mouth and nose, a flat and asymmetric nose, and dental abnormalities, whereas those with palatal involvement can present with speech difficulties, namely hypernasality and articulation difficulties (Hagberg et al., 2019). Surgery to repair the cleft begins within the first year of life, and the main goal of surgery is to correct orofacial form and function. Surgery aims to close the gap in the lip with minimal facial scarring, to minimize the impact upon the symmetry and shape of the nose, and to close the gap in the palate to support normal speech development. Despite multiple surgeries throughout childhood, young people with CL/P may have residual visible facial differences and impaired speech.

### Appearance and Speech Satisfaction

A plethora of research has investigated the psychosocial impact of CL/P (Hunt et al., 2006; Sischo et al., 2017; Sundell et al., 2017). The findings from two systematic reviews (Hunt et al., 2005; Al-Namankany and Alhubaishi, 2018) and one narrative review (Stock and Feragen, 2016) indicate that two key issues that often emerge are dissatisfaction with facial appearance and speech. While some research has found no difference in appearance satisfaction between cleft and noncleft groups (Albers et al., 2016), or even higher appearance satisfaction in the former group (Berger and Dalton, 2009), young people with CL/P often report lower appearance and speech satisfaction compared to noncleft control groups (Hunt et al., 2006; Van Lierde et al., 2012; Wehby et al., 2012). Appearance and speech dissatisfaction can impact several major domains of psychosocial functioning, including self-esteem, mental health, and quality of life (Stock and Feragen, 2016). In fact, many psychosocial difficulties reported in cleft research seem to be related to concerns with appearance and speech (Hunt et al., 2005), making it vital for research to investigate which groups within the CL/P population experience the lowest appearance and speech satisfaction. Studies that have made a distinction between visible and nonvisible clefts often report lower appearance satisfaction in the former group compared to the latter (Feragen et al., 2015; Feragen and Stock, 2016b). It is likely that those with palatal involvement would report the lowest speech satisfaction, although no research has compared speech satisfaction across different cleft diagnostic groups.

It may be overly simplistic to conceptualize appearance and speech satisfaction as simply a product of diagnosis. Another important factor may be age. Among young people with CL/P, cross-sectional studies have demonstrated decreasing appearance satisfaction with increasing age (Thomas et al., 1997; age range: 10-20 years; Hunt et al., 2006; age range: 8-21 years), though others have found no effect of age (Oosterkamp et al., 2007; mean age: 28.2 years; Pitak-Arnnop et al., 2011; age range: 15-48 years). The lack of a significant finding in the latter two studies may be a product of the older age of the participants. According to developmental psychology theories, adolescence is characterized by increased salience of physical appearance, the growing importance of the peer group, and the importance of being “normal” (Sanders, 2013; Gaete, 2015). The theory of adolescent egocentrism (Elkind, 1967) suggests that adolescents often believe that others are paying more attention to their appearance and behavior than is actually the case and often focus their thoughts on the self rather than others. These beliefs are postulated to produce increased self-consciousness in adolescents (Elkind, 1967, 1978). This theoretical framework may explain the decreasing appearance satisfaction observed from preadolescence into adolescence.

Compared to the plethora of research investigating appearance satisfaction, there has been a paucity of psychological research investigating speech satisfaction among young people with CL/P. Self-consciousness in adolescence as postulated by Elkind (1967, 1978) may apply not only to appearance but also to speech. Also, given the theorized importance of the peer group and forming relationships in adolescence (Collins et al., 2009), difficulties in communication due to speech impairment might conceivably result in young people feeling particularly dissatisfied with their speech during this developmental stage. Interestingly, among young people with CL/P, Gkantidis et al. (2015) reported increasing speech satisfaction with increasing age (age range: 9-33 years). However, the use of such a wide age range may hinder the ability to detect changes across discrete developmental stages, a limitation of much cleft research as noted by Stock and Feragen (2019) who advocate the use of developmentally defined age groups wherever possible. Research that focuses specifically on preadolescence and adolescence has found that older respondents report lower speech satisfaction compared to younger respondents (Hunt et al., 2006; age range: 8-21 years; Glener et al., 2017; age range: 9-17 years), which accords with the developmental theories discussed.

A limitation of previous research is that it tends to investigate the effects of age and diagnosis in isolation which does not afford the opportunity to study interaction effects. Given the theorized self-consciousness that characterizes adolescence, we might expect diagnosis to have a greater effect on appearance and speech satisfaction during adolescence compared to preadolescence as young people become increasingly aware of their facial difference or speech impairment.

### Associations With Psychosocial Difficulties

The importance of studying appearance and speech satisfaction is underpinned by their associations with a number of psychosocial difficulties. Crucially, it is not the objective degree of visible difference or speech impairment that seems to be most strongly related to psychosocial difficulties but rather the individual’s subjective appearance and speech satisfaction (Feragen, 2012). Among children and adolescents with CL/P, lower appearance satisfaction has been shown to be associated with greater self-reported depressive and anxious symptoms (Thomas et al., 1997; Feragen et al., 2010, 2015) as well as greater self-reported social difficulties (Pope and Ward, 1997; Feragen and Borge, 2010). These associations are also present in wider psychological literature. For example, Thompson (2012) attempted to synthesize the plethora of theories that exist within appearance research. As part of his model, negative subjective evaluation of appearance, referred to as valence, is bidirectionally associated with emotional and social difficulties.

While much research has focused on appearance satisfaction, very little research has focused on speech satisfaction and associations with psychosocial difficulties. Two studies demonstrated an association between lower speech satisfaction and greater self-reported depressive and anxious symptoms among children with CL/P (Bickham et al., 2017; Feragen et al., 2017). Feragen et al. (2017) also investigated social difficulties, although they found no association with speech satisfaction at 10 years old. Watterson et al. (2013) had noncleft children aged 8 to 11 rate speech samples and found that, as ratings of nasality increased, social acceptance ratings decreased. Even mild hypernasality was sufficient to lead to increased negative social reactions. Similarly, after hearing speech samples of children with cleft palate, 10-year-old children reflected on the negative social consequences and social exclusion that could be experienced by someone with impaired speech (Nyberg and Havstam, 2016). Therefore, further research is needed investigating speech satisfaction as rated by children with CL/P and associations with social difficulties. For both emotional and social difficulties, no research has investigated whether associations with speech satisfaction exist during adolescence.

The relationships between satisfaction and psychosocial difficulties may differ at different ages. During adolescence, feeling “normal” seems to form an important part of well-being (Elkind, 1967, 1978). For young people with CL/P, their appearance and speech may make them feel very different from their peers, and so lower appearance and speech satisfaction may be more strongly associated with increased emotional and social difficulties during adolescence compared to preadolescence. No study to date has statistically compared these associations at these two discrete developmental periods among young people with CL/P. This is important as dissatisfaction with appearance and speech may be ways in which to identify those at risk of psychosocial difficulties within clinical settings, and this may be particularly prominent at certain points in development, namely adolescence.

### Aims and Predictions

While previous research has identified appearance and speech dissatisfaction as key concerns among the CL/P population, less research has been conducted into which groups may be least satisfied and how these two factors might relate to psychosocial difficulties. The aims of this study are twofold. Firstly, this study aims to investigate differences in appearance and speech satisfaction by diagnosis and age among young people with CL/P. Secondly, this study aims to investigate associations between appearance and speech satisfaction and emotional and social difficulties. Based on existing literature, the predictions of this study are as follows:

1. Appearance satisfaction will be lowest for adolescents with lip involvement.
2. Speech satisfaction will be lowest for adolescents with palatal involvement.
3. Lower appearance and speech satisfaction will be associated with increased emotional and social difficulties.
4. These associations will be stronger during adolescence (15 years old) compared to preadolescence (10 years old).

## Method

### Design

Self-report questionnaires that had been collected between June 2016 and August 2018 within routine clinical practice were analyzed.

### Respondents

As part of a national treatment pathway, all patients with CL/P are invited to attend review appointments at ages 5, 10, 15, and 20 years old with the cleft multidisciplinary team. A number of questionnaires are administered to young people who attend these appointments as part of routine clinical practice. The decision is sometimes made not to administer the questionnaires to young people requiring a translator due to time constraints or to those with global developmental delay or complex syndromes who may struggle with questionnaire completion, such as DiGeorge syndrome. All 10- and 15-year-old self-report questionnaires collected over a 27-month-period at a tertiary pediatric hospital in London were retrospectively accessed, resulting in a sample of 156 young people with CL/P. Consent for the study was not sought as no procedures were performed on patients outside of routine clinical care and all responses were anonymized by a member of the care team before analyses were conducted. Nineteen young people were excluded due to the presence of a genetic syndrome or other medical condition that can affect appearance as it was hypothesized that this could confound the results (1 = Silver-Russell syndrome, 2 = Van der Woude, 2 = orofacial digital syndrome, 1 = Binder syndrome, 1 = hemifacial microsomia, 3 = Stickler syndrome, 9 = Pierre Robin sequence). An additional 7 young people with neurodevelopmental disorders were excluded to ensure sample homogeneity (3 = attention deficit hyperactivity disorder, 4 = autism spectrum disorder). Information regarding the presence of additional diagnoses was obtained via the electronic patient record system. The resulting sample comprised of 130 non-syndromic 10- and 15-year-olds with CL/P (see Table 1).

**Table 1.** Sample Demographics.

Gender	Age	Diagnosis	N
Male	10	CL	10
		CP	5
		CLP	24
	15	CL	7
		CP	6
		CLP	13
Female	10	CL	8
		CP	14
		CLP	11
	15	CL	12
		CP	13
		CLP	7

Abbreviations: CL, cleft lip; CLP, cleft lip and palate; CP, cleft palate.

### Materials

#### The Cleft Hearing, Appearance and Speech Questionnaire

Developed by the Clinical Excellence Network of the Craniofacial Society of Great Britain and Ireland, the *Cleft Hearing, Appearance and Speech Questionnaire* (CHASQ) is a 15-item self-report questionnaire measuring satisfaction with cleft-related and noncleft-related facial features and perceived cleft visibility. Respondents are asked to indicate how happy they are with the aforementioned features by responding on 11-point interval scales ranging from 0 (very unhappy) to 10 (very happy). Higher scores indicate greater satisfaction. Two items were used to measure appearance and speech satisfaction respectively: “How happy are you with how your face looks?” and “How happy are you with your speech?” A single item was used to measure speech satisfaction as this is the only item that pertains to speech. A single item was used to measure appearance satisfaction over the use of a mean score of several items. There is no consensus in the literature with regard to how best to use the CHASQ; some studies use a 4-item version (Feragen et al., 2016), some use a 12-item version (Feragen and Stock, 2016b), while some use single items (Berger and Dalton, 2011). Therefore, as this is the only item pertaining to satisfaction with overall facial appearance as opposed to more specific facial features, a single item was used rather a composite score. For the whole sample, these 2 items were significantly correlated (*r*_s_ = 0.498, *P* < .001).

The psychometric properties of the CHASQ have been confirmed, and it has been shown to be a useful and valid clinical and research tool (Clinical Excellence Network, 2015). In 2 large and representative CL/P samples, the CHASQ was shown to possess good to excellent internal consistency (age 10: α = .89; age 16: α = .75) and satisfactory to good validity (Feragen et al., 2015; Feragen and Stock, 2016b).

#### The Strengths and Difficulties Questionnaire

The Strengths and Difficulties Questionnaire (SDQ, Goodman, 1997) is a 25-item behavioral screening questionnaire relating to psychosocial strengths and difficulties as well as the impact of difficulties on daily life. There are 5 subscales: emotional difficulties, social difficulties, conduct problems, hyperactivity/inattention, and prosocial behavior. Each subscale pertains to 5 items. The first 4 subscales are added together to generate a total difficulties score. Based on previous literature, the emotional and social difficulties subscales were the focus of interest in this study. Example statements pertaining to these 2 subscales, respectively, are as follows: “I worry a lot” and “I have one good friend or more.” Respondents are asked to indicate the degree to which a statement applies to how things have been for them over the last 6 months by responding on 3-point Likert scales ranging from not true, somewhat true, to certainly true. The appropriate items are reversed so that a higher score is indicative of greater difficulties, with 10 being the maximum score possible for each subscale.

The SDQ has been used extensively in psychological research and is a well-validated clinical and research tool (Goodman, 1997, 2001; Goodman et al., 2003). Confirmatory factor analysis has demonstrated support for the 5-factor structure of the SDQ (Boe et al., 2016).

### Statistical Analysis

Data were analyzed using IBM SPSS Statistics version 23. Shapiro-Wilk tests revealed that none of the 4 outcome variables were normally distributed (*P* < .001 for appearance satisfaction, speech satisfaction, emotional difficulties, and social difficulties). Neither square root transformations, logarithmic transformations, nor inverse transformations resulted in normally distributed data. Therefore, the data were analyzed using multivariate analysis of variance (MANOVA) with nonparametric follow-up tests and Spearman rank-order correlations. All other statistical assumptions were checked and met. In order to test for differences between the correlation coefficients from the 2 age groups, Fisher *z* transformations were used.

## Results

### Comparison to Normative Data

To the authors’ knowledge, no general population norms currently exist for the CHASQ. Van Roy et al. (2006) administered the SDQ to a large Norwegian sample and reported age and gender specific means for “preadolescents” (10-13 years old, n = 9707) and “early adolescents” (13-16 years old, *n* = 9387). One-sample *t* tests revealed no significant differences with the 10- and 15-year-olds in the current study, respectively, for the emotional and social difficulties subscales (see Tables 2 and 3).

**Table 2.** Mean Scores for Emotional Difficulties Split by Age and Gender: Comparison to Normative Data (Van Roy et al., 2006).^a^

	Emotional difficulties					
	Mean (SD) normative data		Mean (SD) current study		Effect size (*d*)	
	Boys	Girls	Boys	Girls	Boys	Girls
Pre-adol.	2.2 (1.9)	3.0 (2.2)	2.5 (1.8)	2.5 (2.1)	0.17	−0.23
Early-adol.	2.1 (2.1)	3.2 (2.3)	2.6 (1.8)	3.2 (2.3)	0.27	−0.02

^a^ Pre-adol. refers to 10- to 13-year-olds in the study by Van Roy et al. (2006) and 10-year-olds in the current study. Early-adol. Refers to 13-to 16-year-olds in the study by Van Roy et al. (2006) and 15-year-olds in the current study.

**Table 3.** Mean Scores for Social Difficulties Split by Age and Gender: Comparison to Normative Data (Van Roy et al., 2006).^a^

	Social difficulties					
	Mean (SD) normative data		Mean (SD) current study		Effect size (*d*)	
	Boys	Girls	Boys	Girls	Boys	Girls
Pre-adol.	2.1 (1.8)	1.9 (1.7)	1.9 (2.0)	1.8 (1.8)	−0.11	−0.05
Early-adol.	2.1 (2.0)	1.8 (1.8)	1.7 (1.3)	1.8 (1.8)	−0.34	0.01

^a^ Pre-adol. refers to 10- to 13-year-olds in the study by Van Roy et al. (2006) and 10-year-olds in the current study. Early-adol. Refers to 13- to 16-year-olds in the study by Van Roy et al. (2006) and 15-year-olds in the current study.

No significant gender differences were found with regard to appearance satisfaction, *U*(65,65) = 2005, *Z* = −0.52, *P* = .603, *r* = −.05, speech satisfaction *U*(65,65) = 2043, *Z* = −0.35, *P* = .728, *r* = −.03, emotional difficulties *U*(65,65) = 2004, *Z* = −0.51, *P* = .609, *r* = −.04, or social difficulties *U*(65,65) = 2092.50, *Z* = −0.10, *P* = .924, *r* = −.01. Gender was therefore collapsed in subsequent analyses to protect sample size and thus power.

### Descriptive Statistics

Table 4 presents descriptive statistics for the 4 dependent variables. Children with submucous cleft palate were included in the “cleft palate” group.

**Table 4.** Descriptive Statistics, Mean (Standard Deviation), for Appearance Satisfaction, Speech Satisfaction, Emotional Difficulties, and Social Difficulties.

Outcome	Age 10			Age 15		
	CL (n = 18)	CP (n = 19)	CLP (n = 35)	CL (n = 19)	CP (n = 19)	CLP (n = 20)
Appearance satisfaction	8.44 (2.09)	9.26 (1.56)	7.86 (2.60)	7.84 (2.17)	8.26 (1.91)	6.25 (2.27)
Speech satisfaction	9.33 (1.46)	8.95 (2.22)	8.17 (2.48)	8.89 (2.08)	7.53 (3.15)	7.65 (2.25)
Emotional difficulties	2.33 (1.78)	2.26 (2.08)	2.74 (1.95)	2.26 (2.16)	2.74 (1.88)	3.65 (2.11)
Social difficulties	1.94 (1.66)	1.58 (1.71)	1.94 (2.16)	1.05 (1.13)	1.84 (1.43)	2.30 (1.92)

Abbreviations: CL, cleft lip; CLP, cleft lip and palate; CP, cleft palate.

### Study Aim 1: Differences in Appearance and Speech Satisfaction by Diagnosis and Age

In order to investigate the first study aim, a 2-way between groups MANOVA was conducted. Based on Wilks’ lambda criteria for statistical inference, the results from the first 2 × 3 MANOVA revealed a significant multivariate main effect of diagnosis on the combined dependent variables of appearance and speech satisfaction, *F*(4,248) = 4.87, *P* = .001. The partial eta-squared ($\eta_{\text{p}}^{2}$) value of .07 (95% CI: 0.01-0.13) constitutes a medium effect size (Cohen, 1988). Similarly, a significant multivariate main effect of age was found, *F*(2,123) = 3.91, *P* = .023, of medium effect size, $\eta_{\text{p}}^{2}$ = .06, (95% CI: .00-.15). There was no significant multivariate interaction between diagnosis and age, *F*(4,246) = 0.72, *P* = .576, $\eta_{\text{p}}^{2}$ = .01, (95% CI: .00-.03).

An exploration of the univariate effects for appearance satisfaction revealed a significant main effect of diagnosis, *F*(2,124) = 6.96, *P* = .001, of medium effect size, $\eta_{\text{p}}^{2}$ = .10 (95% CI: .02-.20). The Bonferroni-corrected α level of .017 was used as 3 follow-up comparisons were conducted. Mann-Whitney *U* tests revealed that the CLP group reported significantly lower appearance satisfaction (*Mdn* = 8, range = 1-10) compared to the CP group (*Mdn* = 10, range = 4-10), *U*(55,38) = 697.50, *Z* = −2.82, *P* = .005, *r* = −.25. There were no significant differences between the CLP group and the CL group, *U*(55,37) = 825.50, *Z* = −1.57, *P* = .116, *r* = −.14, nor between the CL group and the CP group, *U*(37,38) = 587.50, *Z* = −1.30, *P* = .193, *r* = −.11, with regard to appearance satisfaction. Continuing the univariate effects for appearance satisfaction, there was also a significant main effect of age, *F*(1,124) = 7.33, *P* = .008, of medium effect size, $\eta_{\text{p}}^{2}$ = .06, (95% CI: .00-.15). Descriptive statistics revealed that the older group were less satisfied with their appearance (*Mdn* = 8, range = 2-10) compared to the younger group (*Mdn* = 10, range: 1-10). There was no significant univariate interaction between diagnosis and age, *F*(2,124) = 0.59, *P* = .558, $\eta_{\text{p}}^{2}$ = .01, (95% CI: .00-.06).

When examining the univariate effects for speech satisfaction, there were no significant main effects of diagnosis, *F*(2,124) = 2.88, *P* = .06, $\eta_{\text{p}}^{2}$ = .04, (95% CI: .00-.12), or age, *F*(1,124) = 3.50, *P* = .064, $\eta_{\text{p}}^{2}$ = .03, (95% CI: .00-.10). There was also no significant univariate interaction between diagnosis and age, *F*(2,124) = 0.53, *P* = .592, $\eta_{\text{p}}^{2}$ = .01, (95% CI: .00-.05).

### Study Aim 2: Associations With Psychosocial Difficulties

Spearman correlations were used to investigate associations between appearance and speech satisfaction and psychosocial difficulties. As can be seen in Table 5, significant associations were found at age 10 between appearance satisfaction and social difficulties as well as speech satisfaction and social difficulties of medium and small effect size, respectively (Cohen, 1992). At age 15, significant associations were found for all 4 correlations which were of medium effect size, except for the association between appearance satisfaction and emotional difficulties which was of large effect size (Cohen, 1992).

**Table 5.** Spearman Rank-Order Correlations for Appearance and Speech Satisfaction and Emotional and Social Difficulties at Age 10 and Age 15.

	Age 10		Age 15	
	Appearance satisfaction	Speech satisfaction	Appearance satisfaction	Speech satisfaction
Emotional difficulties	−.22	−.16	−.54^a^	−.44^b^
Social difficulties	−.30^c^	−.25^c^	−.38^b^	−.33^c^

^a^ *P* < .001.

^b^ *P* < .01.

^c^ *P* < .05.

Fisher *z* transformation was used to test for differences between the correlation coefficients from the 2 age groups. This revealed that the relationship between appearance satisfaction and emotional difficulties was significantly stronger at age 15 compared to age 10 (*z* = 2.12, *P* = .017), as was the relationship between speech satisfaction and emotional difficulties (*z* = 1.71, *P* = .043). No significant differences were found between the 2 age groups with regard to the relationships between appearance satisfaction and social difficulties (*z* = 0.55, *P* = .291) nor speech satisfaction and social difficulties (*z* = 0.51, *P* = .306).

## Discussion

### Differences in Appearance and Speech Satisfaction by Diagnosis and Age

The first study aim was to investigate differences in appearance and speech satisfaction by diagnosis and age among young people with CL/P. The results indicate that those with cleft lip and palate were less satisfied with their appearance compared to those with cleft palate only, although no other differences between diagnostic groups were found. The adolescent group was less satisfied with their appearance compared to the preadolescent group. Therefore, the prediction that appearance satisfaction would be lowest for adolescents with lip involvement was partly supported, although no interaction was found. Conversely, speech satisfaction did not differ across age nor across diagnostic group. Therefore, the prediction that speech satisfaction would be lowest for adolescents with palatal involvement was not supported.

In terms of differences by diagnosis, the lack of difference in appearance satisfaction between those with cleft lip and those with cleft palate is surprising. Similarly, it is interesting that speech satisfaction was comparable across all diagnostic groups. Interestingly, scores on the CHASQ were generally high and therefore perhaps there were few differences in appearance and speech satisfaction by diagnosis because respondents were reasonably satisfied with appearance and speech. The findings are therefore important in questioning the assumption that visible facial difference and speech impairment inevitably lead to dissatisfaction with appearance and speech.

With regard to age effects, the lower appearance satisfaction of the adolescent group compared to the preadolescent group is consistent with previous research (Thomas et al., 1997; Hunt et al., 2006) and supports theories of adolescence delineating increased salience of physical appearance and increased self-consciousness during this developmental stage (Elkind, 1967, 1978). Therefore, lower appearance satisfaction may be a product of normative developmental changes. Furthermore, given the importance attributed to being “normal” during adolescence, appearance dissatisfaction may be exacerbated in the CL/P population where visible facial differences may contribute to a feeling of being different from one’s peers. Indeed, Glener et al. (2017) discuss the increased aesthetic concerns of adolescents with CL/P compared to preadolescents with CL/P in the context of the stigmatization of being different. In contrast, speech satisfaction did not differ according to age, suggesting that increased self-consciousness as theorized by Elkind (1967, 1978) may apply to appearance but not to speech. It is also possible that treatment-related changes could contribute to changes in appearance and speech satisfaction over time, such as the commencement of orthodontic treatment. In a similar vein, the speech profiles of the sample and impact of any speech therapy should be considered in future research given the impact this may have on speech satisfaction.

### Associations With Psychosocial Difficulties

The second study aim was to investigate associations between appearance and speech satisfaction and emotional and social difficulties. The results indicate that lower appearance and speech satisfaction are associated with increased emotional and social difficulties, although these associations were only all significant at age 15 and not age 10, thus partly supporting the prediction of negative associations between these variables. Furthermore, the associations for emotional difficulties, but not social difficulties, were significantly stronger at age 15 compared to age 10, partly supporting the prediction that associations would be stronger during adolescence compared to preadolescence.

The associations found between appearance satisfaction and emotional and social difficulties are consistent with previous research (Thompson, 2012; Feragen et al., 2015), with the exception of the lack of a significant association between appearance satisfaction and emotional difficulties at age 10. Perhaps appearance concerns are not as salient to preadolescents and thus are not consistently associated with emotional difficulties. In the current study, a large effect size was found for the relationship between appearance satisfaction and emotional difficulties at age 15. This is especially noteworthy in the context of researchers arguing that the value delineated by Cohen (1992) to indicate a large correlation coefficient (.5) occurs infrequently in psychological research and so a lower value may be more appropriate (Hemphill, 2003). This relationship was also significantly stronger at age 15 compared to age 10, which supports research suggesting that appearance concerns may be a particularly important factor related to increased emotional difficulties during adolescence (Graber and Sontag, 2009). It is surprising that the relationship between appearance satisfaction and social difficulties did not differ significantly at the two ages given that appearance seems to be strongly related to social concerns, social status, and forming relationships during adolescence (Kierans and Swords, 2016).

With regard to the associations for speech, the lack of a significant association between speech satisfaction and emotional difficulties at age 10 is inconsistent with previous cleft research (Bickham et al., 2017; Feragen et al., 2017). Bickham et al. (2017) focused specifically on depression and so perhaps by measuring “emotional difficulties,” a construct incorporating both depressive and anxious symptoms, this may have resulted in a nonsignificant association in the current study. However, Feragen et al. (2017) similarly used the emotional difficulties subscale of the SDQ with 10-year-old children and did find a significant association with speech satisfaction. Conversely, these authors failed to find a significant association between speech satisfaction and social difficulties at age 10, which was found in the current study, although this was of small effect size. Nevertheless, in the current study, significant associations were found at 15 years old for both emotional and social difficulties, which adds to existing research as no research had previously been conducted using adolescents. The relationship between speech satisfaction and emotional difficulties was also significantly stronger at age 15 compared to age 10, which suggests that speech satisfaction may be an important factor related to increased emotional difficulties during adolescence.

### Study Limitations

There are several limitations of the current study. Firstly, there are limitations regarding the use of the CHASQ. Lack of comparison to a noncleft control group hinders the interpretation of the findings. It is unclear whether diagnostic groups were similarly happy with appearance and speech or similarly unhappy as, without norms, it is difficult to establish what constitutes a “high” score on the CHASQ. Also, without norms, it is difficult to distinguish between condition-specific and general population findings (Stock et al., 2018). It may be that, among young people with CL/P, appearance satisfaction declines in adolescence in the same way as the general population. Alternatively, it may be that a sharper decline is experienced among the CL/P population due to the stigmatization of being different that may be exacerbated during adolescence, when the importance of “fitting in” is heightened. Furthermore, using single items to capture the complex and multifaceted constructs of appearance and speech satisfaction may be overly simplistic. There are many aspects to speech in young people with CL/P, including speech sound production, resonance, and perceived intelligibility. Future investigation of appearance and speech satisfaction may benefit from more comprehensive, composite measures that account for the many different aspects of appearance and speech about which young people with CL/P may feel satisfied or dissatisfied.

Another methodological limitation is the exclusion of those with genetic syndromes and neurodevelopmental disorders. Although this was done to ensure sample homogeneity, the widespread exclusion of individuals with additional diagnoses in cleft research means that this subgroup is poorly understood, despite comprising a substantial proportion of the CL/P population and being potentially the most vulnerable (Feragen and Stock, 2014). In future research, an inclusive approach may be more clinically useful whereby subgroups are clearly defined rather than excluded. Unfortunately, the sample size was not large enough to permit such an approach in this study.

A final limitation of this study is the correlational and cross-sectional design which makes it difficult to establish the direction of relationships between appearance and speech satisfaction and psychosocial difficulties. It may be that appearance and speech dissatisfaction play a role in the development of psychosocial difficulties, the converse may be true, or it may be that bidirectional relationships exist between these variables whereby they serve to exacerbate one another over time. Furthermore, such a design results in the possibility that relationships are influenced by confounding variables. For example, bullying contributes to negative self-perceptions as well as to emotional and social difficulties (Feragen, 2012) and so could partly account for the relationships found in the current study. One study found that 30% of CL/P children reported being bullied because of their cleft and this increased to 60% among CL/P adolescents (Nicholls et al., 2019), with appearance and speech often being reported as the reason for bullying (Lorot-Marchand et al., 2015; Bhat et al., 2019; Nicholls et al., 2019). Therefore, future research might usefully investigate the role of bullying in moderating the relationships between appearance and speech satisfaction and psychosocial difficulties.

### Implications for Future Research

In terms of implications for future research, studies using a longitudinal cross-lagged panel design are needed in order to elucidate the direction of associations between appearance and speech satisfaction and psychosocial difficulties and to investigate whether these relationships are moderated by other variables. Among young people with CL/P, Feragen and Stock (2016a) found that teasing after 10 years old can lead to appearance dissatisfaction and emotional difficulties during adolescence, supporting the need for further longitudinal studies investigating the temporal relationships between these variables. Qualitative research would also be useful in exploring appearance and speech satisfaction in more depth among the CL/P population, particularly among adolescents. Using a mixed-methods design, Griffiths et al. (2012) explored the romantic experiences of adolescents with a visible difference. The qualitative component allowed researchers to obtain a rich understanding of how appearance-related concerns can cause distress among this group due to fear of negative evaluation by potential partners.

A further consideration that is relevant to future research is the assumption that negative experiences will ensue from having a visible facial difference or impaired speech. This assumption has contributed to a problem-focused approach to cleft research whereby difficulties and problems are prioritized at the expense of researching positive coping and resilience. Feragen et al. (2009) investigated psychosocial resilience in 10-year-old CL/P children and found that decreased teasing, questions, and staring differentiated the resilient from the nonresilient children, as well as higher appearance and speech satisfaction. Furthermore, qualitative studies have revealed several positive outcomes of living with a facial difference, such as greater personal strength, increased appreciation of diversity, and an ability to cope with challenges (Eiserman, 2001; Meyerson, 2001). Therefore, a more balanced approach is needed in future cleft research whereby equal consideration is given to psychosocial resilience and psychosocial difficulties.

### Implications for Clinical Practice

This study adds to the existing evidence base that dissatisfaction with appearance and speech may be ways in which to identify young people with CL/P at risk of psychosocial difficulties within clinical settings. Adolescence seems to be a particularly vulnerable point in development where appearance satisfaction may be lowest and where appearance and speech dissatisfaction may be most strongly associated with increased emotional difficulties.

The findings underscore the vital role of clinical psychologists in cleft care. Although caution must be taken in interpreting CHASQ scores due to the lack of normative data, it does seem as though the current sample were, on average, reasonably satisfied with appearance and speech. At the point where young people stop growing, the possibility of having elective surgery to alter appearance or speech is made available to patients. Although some young people may request further surgery in the hope of altering nasal appearance or improving speech quality, it should not be assumed that all young people with CL/P are dissatisfied with appearance and speech. The few differences found in appearance and speech satisfaction by diagnosis clearly indicate that the presence of a visible facial difference or speech impairment alone is insufficient to understand who may be least satisfied with appearance and speech. This conclusion is supported by the negligible associations found between professional evaluation and patient satisfaction with appearance and speech (Feragen et al., 2017; Mulder et al., 2019). Therefore, conversations about further surgery should always be guided by the individual patient in collaboration with the psychologist who may be best placed to assess desire and expectations for surgery, as well as overall psychosocial well-being in relation to appearance and speech.

## Conclusion

In summary, among the CL/P population, adolescents as well as those with cleft lip and palate may experience the lowest appearance satisfaction, whereas speech satisfaction does not seem to differ according to diagnosis or age. Lower appearance and speech satisfaction seems to be associated with increased emotional and social difficulties, and associations with emotional difficulties seem to be stronger during adolescence compared to preadolescence. Although it is important to consider the psychosocial difficulties that young people with CL/P may experience, it is equally important to consider psychosocial resilience among this population, an often-neglected construct in cleft research. In the study by Eiserman (2001), adults were asked, if given the choice, would they remove the facial difference experience from their lives? In an eloquent response that powerfully refutes the assumption of negative experiences ensuing from a CL/P diagnosis, one adult with CL/P responded:

Earlier in my life I would have answered this question with a strong, “Yes.” I would have viewed the chance to get rid of my cleft like “shedding a skin.” Today I think it would feel more like losing a limb since it’s been such an integral part of my life. (Eiserman, 2001, p241).

## Research Data

Research Data abstract for Appearance and Speech Satisfaction and Their Associations With Psychosocial Difficulties Among Young People With Cleft Lip and/or Palate Click here for additional data file. Research Data abstract for Appearance and Speech Satisfaction and Their Associations With Psychosocial Difficulties Among Young People With Cleft Lip and/or Palate by Sarah N. Kelly and Joanna Shearer in The Cleft Palate-Craniofacial Journal This article is distributed under the terms of the Creative Commons Attribution 4.0 License ( https://creativecommons.org/licenses/by/4.0/) which permits any use, reproduction and distribution of the work without further permission provided the original work is attributed as specified on the SAGE and Open Access page ( https://us.sagepub.com/en-us/nam/open-access-at-sage).
