# Supplementary material for: Appearance and Speech Satisfaction and Their Associations With Psychosocial Difficulties Among Young People With Cleft Lip and/or Palate
Source: Cleft Palate Craniofac J. 2020 May 28;57(8):1008–17. doi: 10.1177/1055665620926083 (PMC7361652; doi:10.1177/1055665620926083)
Supplement: Research Data abstract for Appearance and Speech Satisfaction and Their Associations With Psychosocial Difficulties Among Young People With Cleft Lip and/or Palate [file Research_Data.docx]

| Gender | Diagnosis | Additional_diagnoses | What_additional_diagnoses | Age |
| --- | --- | --- | --- | --- |
| 1 | 1 | 1 | No | 10 |
| 2 | 1 | 1 | No | 10 |
| 1 | 1 | 1 | No | 10 |
| 1 | 1 | 1 | No | 10 |
| 2 | 1 | 1 | No | 10 |
| 2 | 1 | 1 | No | 10 |
| 2 | 1 | 1 | No | 10 |
| 1 | 1 | 1 | No | 10 |
| 2 | 1 | 1 | No | 10 |
| 1 | 1 | 1 | No | 10 |
| 1 | 1 | 1 | No | 10 |
| 1 | 1 | 1 | No | 10 |
| 2 | 1 | 1 | No | 10 |
| 1 | 1 | 1 | No | 10 |
| 2 | 1 | 1 | No | 10 |
| 2 | 1 | 1 | No | 10 |
| 1 | 1 | 1 | No | 10 |
| 1 | 1 | 1 | No | 10 |
| 2 | 1 | 1 | No | 15 |
| 2 | 1 | 1 | No | 15 |
| 2 | 1 | 1 | No | 15 |
| 2 | 1 | 1 | No | 15 |
| 1 | 1 | 1 | No | 15 |
| 2 | 1 | 1 | No | 15 |
| 2 | 1 | 1 | No | 15 |
| 2 | 1 | 1 | No | 15 |
| 2 | 1 | 1 | No | 15 |
| 1 | 1 | 1 | No | 15 |
| 2 | 1 | 1 | No | 15 |
| 1 | 1 | 1 | No | 15 |
| 2 | 1 | 1 | No | 15 |
| 2 | 1 | 1 | No | 15 |
| 2 | 1 | 1 | No | 15 |
| 1 | 1 | 1 | No | 15 |
| 1 | 1 | 1 | No | 15 |
| 1 | 1 | 1 | No | 15 |
| 1 | 1 | 1 | No | 15 |
| 2 | 2 | 1 | No | 10 |
| 1 | 2 | 1 | No | 10 |
| 2 | 2 | 1 | No | 10 |
| 2 | 2 | 1 | No | 10 |
| 1 | 2 | 1 | No | 10 |
| 2 | 2 | 1 | No | 10 |
| 1 | 2 | 1 | No | 10 |
| 1 | 2 | 1 | No | 10 |
| 2 | 2 | 1 | No | 10 |
| 2 | 2 | 1 | No | 10 |
| 2 | 2 | 1 | No | 10 |
| 2 | 2 | 1 | No | 10 |
| 2 | 2 | 1 | No | 10 |
| 2 | 2 | 1 | No | 10 |
| 2 | 2 | 1 | No | 10 |
| 2 | 2 | 1 | No | 10 |
| 2 | 2 | 1 | No | 10 |
| 1 | 2 | 1 | No | 10 |
| 2 | 2 | 1 | No | 10 |
| 2 | 2 | 2 | Russell-Silver syndrome | 10 |
| 2 | 2 | 2 | Orofacial digital syndrome | 10 |
| 1 | 2 | 2 | PRS | 10 |
| 2 | 2 | 2 | PRS | 10 |
| 2 | 2 | 2 | ASD | 10 |
| 1 | 2 | 2 | PRS | 10 |
| 1 | 2 | 2 | ADHD | 10 |
| 2 | 2 | 2 | Hemifacial microsomia, PRS | 10 |
| 2 | 2 | 2 | PRS | 10 |
| 2 | 2 | 2 | PRS | 10 |
| 2 | 2 | 2 | PRS | 10 |
| 2 | 2 | 2 | PRS, stickler syndrome | 10 |
| 2 | 2 | 1 | No | 15 |
| 2 | 2 | 1 | No | 15 |
| 1 | 2 | 1 | No | 15 |
| 2 | 2 | 1 | No | 15 |
| 2 | 2 | 1 | No | 15 |
| 1 | 2 | 1 | No | 15 |
| 2 | 2 | 1 | No | 15 |
| 1 | 2 | 1 | No | 15 |
| 2 | 2 | 1 | No | 15 |
| 1 | 2 | 1 | No | 15 |
| 1 | 2 | 1 | No | 15 |
| 2 | 2 | 1 | No | 15 |
| 1 | 2 | 1 | No | 15 |
| 2 | 2 | 1 | No | 15 |
| 2 | 2 | 1 | No | 15 |
| 2 | 2 | 1 | No | 15 |
| 2 | 2 | 1 | No | 15 |
| 2 | 2 | 1 | No | 15 |
| 2 | 2 | 1 | No | 15 |
| 1 | 2 | 2 | PRS, ADHD | 15 |
| 1 | 2 | 2 | ASD | 15 |
| 1 | 2 | 2 | PRS, stickler syndrome | 15 |
| 2 | 2 | 2 | Stickler syndrome | 15 |
| 1 | 2 | 2 | ASD | 15 |
| 2 | 2 | 2 | PRS | 15 |
| 2 | 2 | 2 | PRS | 15 |
| 1 | 2 | 2 | PRS | 15 |
| 2 | 2 | 2 | Van de Woude syndrome | 15 |
| 2 | 2 | 2 | Orofacial digital syndrome | 15 |
| 1 | 3 | 1 | No | 10 |
| 2 | 3 | 1 | No | 10 |
| 2 | 3 | 1 | No | 10 |
| 1 | 3 | 1 | No | 10 |
| 1 | 3 | 1 | No | 10 |
| 2 | 3 | 1 | No | 10 |
| 1 | 3 | 1 | No | 10 |
| 1 | 3 | 1 | No | 10 |
| 1 | 3 | 1 | No | 10 |
| 1 | 3 | 1 | No | 10 |
| 1 | 3 | 1 | No | 10 |
| 2 | 3 | 1 | No | 10 |
| 1 | 3 | 1 | No | 10 |
| 2 | 3 | 1 | No | 10 |
| 1 | 3 | 1 | No | 10 |
| 1 | 3 | 1 | No | 10 |
| 2 | 3 | 1 | No | 10 |
| 1 | 3 | 1 | No | 10 |
| 2 | 3 | 1 | No | 10 |
| 1 | 3 | 1 | No | 10 |
| 1 | 3 | 1 | No | 10 |
| 1 | 3 | 1 | No | 10 |
| 1 | 3 | 1 | No | 10 |
| 1 | 3 | 1 | No | 10 |
| 1 | 3 | 1 | No | 10 |
| 1 | 3 | 1 | No | 10 |
| 2 | 3 | 1 | No | 10 |
| 1 | 3 | 1 | No | 10 |
| 2 | 3 | 1 | No | 10 |
| 2 | 3 | 1 | No | 10 |
| 2 | 3 | 1 | No | 10 |
| 1 | 3 | 1 | No | 10 |
| 1 | 3 | 1 | No | 10 |
| 1 | 3 | 1 | No | 10 |
| 1 | 3 | 1 | No | 10 |
| 1 | 3 | 2 | Van de Woude syndrome | 10 |
| 1 | 3 | 2 | Binder syndrome | 10 |
| 1 | 3 | 1 | No | 15 |
| 2 | 3 | 1 | No | 15 |
| 1 | 3 | 1 | No | 15 |
| 2 | 3 | 1 | No | 15 |
| 1 | 3 | 1 | No | 15 |
| 1 | 3 | 1 | No | 15 |
| 1 | 3 | 1 | No | 15 |
| 1 | 3 | 1 | No | 15 |
| 2 | 3 | 1 | No | 15 |
| 1 | 3 | 1 | No | 15 |
| 1 | 3 | 1 | No | 15 |
| 2 | 3 | 1 | No | 15 |
| 1 | 3 | 1 | No | 15 |
| 1 | 3 | 1 | No | 15 |
| 1 | 3 | 1 | No | 15 |
| 1 | 3 | 1 | No | 15 |
| 2 | 3 | 1 | No | 15 |
| 2 | 3 | 1 | No | 15 |
| 1 | 3 | 1 | No | 15 |
| 2 | 3 | 1 | No | 15 |
| 1 | 3 | 2 | ADHD | 15 |
| 1 | 3 | 2 | ASD | 15 |

| CHASQ_face_looks | CHASQ_speech | SDQ_emotional | SDQ_peer_problems |
| --- | --- | --- | --- |
| 10 | 8 | 5 | 0 |
| 10 | 10 | 2 | 2 |
| 5 | 10 | 5 | 0 |
| 10 | 10 | 2 | 2 |
| 7 | 10 | 0 | 1 |
| 10 | 9 | 4 | 1 |
| 9 | 9 | 0 | 2 |
| 10 | 10 | 1 | 2 |
| 5 | 10 | 2 | 4 |
| 10 | 9 | 1 | 1 |
| 8 | 10 | 2 | 2 |
| 4 | 4 | 5 | 7 |
| 10 | 10 | 1 | 0 |
| 7 | 10 | 5 | 3 |
| 10 | 10 | 0 | 3 |
| 7 | 9 | 3 | 2 |
| 10 | 10 | 2 | 2 |
| 10 | 10 | 2 | 1 |
| 10 | 10 | 1 | 0 |
| 6 | 10 | 1 | 1 |
| 8 | 10 | 0 | 2 |
| 8 | 9 | 4 | 0 |
| 10 | 10 | 2 | 0 |
| 7 | 10 | 3 | 0 |
| 2 | 2 | 6 | 3 |
| 8 | 9 | 0 | 0 |
| 8 | 10 | 1 | 1 |
| 10 | 10 | 3 | 3 |
| 5 | 10 | 5 | 1 |
| 9 | 7 | 0 | 1 |
| 10 | 7 | 5 | 1 |
| 8 | 10 | 1 | 0 |
| 7 | 9 | 6 | 2 |
| 10 | 10 | 0 | 0 |
| 8 | 6 | 1 | 3 |
| 5 | 10 | 4 | 2 |
| 10 | 10 | 0 | 0 |
| 9 | 9 | 0 | 3 |
| 10 | 9 | 0 | 0 |
| 10 | 10 | 4 | 0 |
| 10 | 6 | 2 | 1 |
| 10 | 10 | 1 | 0 |
| 10 | 10 | 1 | 1 |
| 10 | 10 | 1 | 2 |
| 10 | 10 | 1 | 2 |
| 10 | 10 | 5 | 2 |
| 5 | 7 | 5 | 7 |
| 9 | 9 | 4 | 3 |
| 10 | 10 | 3 | 1 |
| 9 | 9 | 0 | 2 |
| 10 | 10 | 0 | 0 |
| 5 | 10 | 4 | 1 |
| 10 | 10 | 1 | 0 |
| 10 | 10 | 1 | 0 |
| 10 | 10 | 3 | 2 |
| 9 | 1 | 7 | 3 |
| 10 | 10 | 6 | 7 |
| 10 | 10 | 2 | 4 |
| 10 | 8 | 3 | 0 |
| 6 | 3 | 5 | 5 |
| 9 | 10 | 5 | 4 |
| 9 | 8 | 9 | 1 |
| 10 | 4 | 0 | 4 |
| 8 | 5 | 1 | 0 |
| 10 | 10 | 9 | 8 |
| 8 | 7 | 2 | 2 |
| 10 | 10 | 1 | 2 |
| 10 | 10 | 3 | 2 |
| 4 | 10 | 4 | 2 |
| 7 | 8 | 4 | 1 |
| 10 | 10 | 4 | 4 |
| 8 | 0 | 4 | 2 |
| 10 | 10 | 0 | 0 |
| 9 | 7 | 3 | 0 |
| 10 | 10 | 1 | 4 |
| 10 | 10 | 0 | 1 |
| 9 | 9 | 0 | 0 |
| 5 | 5 | 2 | 2 |
| 8 | 10 | 2 | 1 |
| 9 | 7 | 1 | 4 |
| 5 | 3 | 5 | 3 |
| 10 | 10 | 4 | 2 |
| 8 | 10 | 5 | 4 |
| 8 | 2 | 6 | 2 |
| 10 | 7 | 2 | 1 |
| 10 | 10 | 1 | 0 |
| 7 | 5 | 4 | 2 |
| 6 | 4 | 4 | 3 |
| 10 | 10 | 4 | 4 |
| 7 | 3 | 3 | 1 |
| 10 | 10 | 5 | 1 |
| 8 | 2 | 5 | 7 |
| 10 | 8 | 2 | 0 |
| 7 | 6 | 5 | 1 |
| 4 | 5 | 8 | 4 |
| 3 | 6 | 3 | 2 |
| 9 | 5 | 9 | 4 |
| 8 | 6 | 5 | 4 |
| 7 | 9 | 3 | 0 |
| 10 | 10 | 7 | 7 |
| 10 | 10 | 2 | 0 |
| 5 | 8 | 2 | 0 |
| 7 | 9 | 1 | 1 |
| 10 | 10 | 2 | 0 |
| 4 | 4 | 1 | 2 |
| 2 | 9 | 4 | 8 |
| 9 | 10 | 0 | 0 |
| 10 | 9 | 2 | 1 |
| 10 | 10 | 2 | 3 |
| 1 | 5 | 3 | 1 |
| 9 | 9 | 3 | 1 |
| 5 | 10 | 1 | 2 |
| 10 | 10 | 3 | 1 |
| 5 | 3 | 3 | 2 |
| 8 | 7 | 1 | 3 |
| 3 | 5 | 3 | 2 |
| 5 | 0 | 8 | 4 |
| 10 | 10 | 3 | 0 |
| 9 | 8 | 0 | 1 |
| 10 | 10 | 0 | 0 |
| 10 | 10 | 5 | 1 |
| 9 | 10 | 3 | 1 |
| 6 | 10 | 4 | 2 |
| 10 | 5 | 2 | 1 |
| 10 | 7 | 3 | 6 |
| 7 | 9 | 3 | 0 |
| 10 | 10 | 0 | 0 |
| 10 | 10 | 7 | 4 |
| 9 | 10 | 4 | 3 |
| 8 | 7 | 1 | 0 |
| 10 | 10 | 3 | 1 |
| 9 | 7 | 2 | 6 |
| 10 | 6 | 0 | 1 |
| 5 | 5 | 4 | 2 |
| 8 | 10 | 4 | 1 |
| 7 | 7 | 6 | 1 |
| 10 | 10 | 2 | 0 |
| 3 | 2 | 3 | 1 |
| 8 | 7 | 4 | 4 |
| 10 | 10 | 1 | 2 |
| 10 | 10 | 0 | 1 |
| 6 | 8 | 1 | 2 |
| 6 | 8 | 4 | 4 |
| 5 | 7 | 4 | 2 |
| 4 | 9 | 3 | 2 |
| 2 | 7 | 6 | 8 |
| 5 | 9 | 4 | 3 |
| 5 | 7 | 5 | 3 |
| 7 | 7 | 5 | 0 |
| 6 | 3 | 5 | 3 |
| 5 | 9 | 9 | 4 |
| 8 | 10 | 1 | 1 |
| 6 | 8 | 3 | 0 |
| 4 | 5 | 3 | 4 |
| 4 | 4 | 8 | 3 |
| 7 | 9 | 2 | 4 |

| SDQ_hyperactivity | SDQ_conduct | SDQ_prosocial | SDQ_total_difficulties | filter_$ |
| --- | --- | --- | --- | --- |
| 7 | 2 | 8 | 14 | 1 |
| 0 | 0 | 10 | 4 | 1 |
| 8 | 5 | 6 | 18 | 1 |
| 7 | 2 | 4 | 13 | 1 |
| 0 | 0 | 10 | 1 | 1 |
| 1 | 1 | 8 | 7 | 1 |
| 3 | 3 | 10 | 8 | 1 |
| 0 | 2 | 10 | 5 | 1 |
| 7 | 3 | 9 | 16 | 1 |
| 6 | 6 | 6 | 14 | 1 |
| 1 | 2 | 8 | 7 | 1 |
| 2 | 3 | 6 | 17 | 1 |
| 4 | 0 | 10 | 5 | 1 |
| 5 | 1 | 4 | 14 | 1 |
| 0 | 0 | 9 | 3 | 1 |
| 4 | 2 | 7 | 11 | 1 |
| 1 | 2 | 7 | 7 | 1 |
| 3 | 4 | 10 | 10 | 1 |
| 5 | 2 | 7 | 8 | 1 |
| 2 | 2 | 7 | 6 | 1 |
| 3 | 1 | 9 | 6 | 1 |
| 4 | 1 | 10 | 9 | 1 |
| 7 | 1 | 8 | 10 | 1 |
| 0 | 0 | 10 | 3 | 1 |
| 6 | 2 | 8 | 17 | 1 |
| 1 | 0 | 9 | 1 | 1 |
| 1 | 1 | 9 | 4 | 1 |
| 7 | 8 | 4 | 21 | 1 |
| 5 | 2 | 8 | 13 | 1 |
| 1 | 0 | 8 | 2 | 1 |
| 3 | 2 | 8 | 11 | 1 |
| 2 | 0 | 9 | 3 | 1 |
| 1 | 0 | 7 | 9 | 1 |
| 6 | 3 | 8 | 9 | 1 |
| 2 | 0 | 4 | 6 | 1 |
| 4 | 1 | 9 | 11 | 1 |
| 2 | 1 | 8 | 3 | 1 |
| 2 | 0 | 7 | 5 | 1 |
| 2 | 5 | 7 | 7 | 1 |
| 0 | 1 | 10 | 5 | 1 |
| 8 | 4 | 6 | 15 | 1 |
| 1 | 1 | 10 | 3 | 1 |
| 4 | 1 | 8 | 7 | 1 |
| 4 | 5 | 8 | 12 | 1 |
| 3 | 2 | 10 | 8 | 1 |
| 0 | 3 | 8 | 10 | 1 |
| 8 | 3 | 8 | 23 | 1 |
| 4 | 3 | 7 | 14 | 1 |
| 4 | 2 | 9 | 10 | 1 |
| 4 | 0 | 9 | 6 | 1 |
| 0 | 0 | 9 | 0 | 1 |
| 9 | 3 | 10 | 17 | 1 |
| 0 | 0 | 8 | 1 | 1 |
| 0 | 0 | 9 | 1 | 1 |
| 3 | 1 | 7 | 9 | 1 |
| 5 | 4 | 5 | 19 | 1 |
| 8 | 2 | 9 | 23 | 0 |
| 2 | 3 | 10 | 11 | 0 |
| 6 | 2 | 9 | 11 | 0 |
| 4 | 2 | 10 | 16 | 0 |
| 4 | 3 | 9 | 16 | 0 |
| 7 | 2 | 6 | 19 | 0 |
| 10 | 6 | 6 | 20 | 0 |
| 5 | 3 | 7 | 9 | 0 |
| 6 | 4 | 10 | 27 | 0 |
| 6 | 4 | 9 | 14 | 0 |
| 4 | 2 | 10 | 9 | 0 |
| 1 | 1 | 9 | 7 | 0 |
| 3 | 1 | 10 | 10 | 1 |
| 5 | 2 | 8 | 12 | 1 |
| 5 | 1 | 10 | 14 | 1 |
| 4 | 1 | 9 | 11 | 1 |
| 0 | 0 | 10 | 0 | 1 |
| 6 | 5 | 7 | 14 | 1 |
| 0 | 0 | 9 | 5 | 1 |
| 2 | 2 | 8 | 5 | 1 |
| 0 | 0 | 10 | 0 | 1 |
| 5 | 4 | 8 | 13 | 1 |
| 5 | 0 | 9 | 8 | 1 |
| 2 | 3 | 7 | 10 | 1 |
| 6 | 1 | 6 | 15 | 1 |
| 4 | 1 | 10 | 11 | 1 |
| 2 | 1 | 7 | 12 | 1 |
| 6 | 0 | 10 | 14 | 1 |
| 2 | 0 | 8 | 5 | 1 |
| 0 | 1 | 10 | 2 | 1 |
| 3 | 1 | 7 | 10 | 1 |
| 8 | 3 | 8 | 18 | 0 |
| 3 | 2 | 4 | 13 | 0 |
| 9 | 1 | 5 | 14 | 0 |
| 4 | 0 | 7 | 10 | 0 |
| 10 | 7 | 7 | 29 | 0 |
| 2 | 0 | 10 | 4 | 0 |
| 2 | 0 | 8 | 8 | 0 |
| 7 | 3 | 9 | 22 | 0 |
| 2 | 1 | 8 | 8 | 0 |
| 6 | 3 | 10 | 22 | 0 |
| 5 | 3 | 9 | 17 | 1 |
| 5 | 0 | 9 | 8 | 1 |
| 5 | 4 | 10 | 23 | 1 |
| 6 | 2 | 10 | 10 | 1 |
| 6 | 2 | 10 | 10 | 1 |
| 4 | 2 | 9 | 8 | 1 |
| 0 | 0 | 10 | 2 | 1 |
| 4 | 3 | 5 | 10 | 1 |
| 7 | 2 | 6 | 21 | 1 |
| 3 | 1 | 7 | 4 | 1 |
| 4 | 3 | 6 | 10 | 1 |
| 2 | 2 | 9 | 9 | 1 |
| 7 | 5 | 6 | 16 | 1 |
| 5 | 1 | 9 | 10 | 1 |
| 2 | 0 | 9 | 5 | 1 |
| 4 | 2 | 8 | 10 | 1 |
| 2 | 1 | 10 | 8 | 1 |
| 3 | 1 | 8 | 8 | 1 |
| 4 | 2 | 8 | 11 | 1 |
| 9 | 5 | 9 | 26 | 1 |
| 4 | 2 | 8 | 9 | 1 |
| 2 | 2 | 8 | 5 | 1 |
| 1 | 3 | 7 | 4 | 1 |
| 1 | 2 | 10 | 9 | 1 |
| 1 | 0 | 9 | 5 | 1 |
| 3 | 1 | 10 | 10 | 1 |
| 3 | 0 | 10 | 6 | 1 |
| 2 | 1 | 6 | 12 | 1 |
| 6 | 1 | 10 | 10 | 1 |
| 0 | 1 | 10 | 1 | 1 |
| 7 | 4 | 10 | 22 | 1 |
| 6 | 0 | 6 | 13 | 1 |
| 3 | 2 | 8 | 6 | 1 |
| 3 | 1 | 7 | 8 | 1 |
| 4 | 2 | 9 | 14 | 1 |
| 4 | 1 | 6 | 6 | 0 |
| 0 | 2 | 9 | 8 | 0 |
| 9 | 4 | 5 | 18 | 1 |
| 6 | 1 | 10 | 14 | 1 |
| 3 | 0 | 8 | 5 | 1 |
| 3 | 1 | 10 | 8 | 1 |
| 3 | 0 | 5 | 11 | 1 |
| 1 | 1 | 5 | 5 | 1 |
| 2 | 1 | 8 | 4 | 1 |
| 3 | 2 | 10 | 8 | 1 |
| 5 | 4 | 5 | 17 | 1 |
| 3 | 2 | 10 | 11 | 1 |
| 2 | 1 | 6 | 8 | 1 |
| 2 | 1 | 9 | 19 | 1 |
| 4 | 4 | 8 | 15 | 1 |
| 4 | 1 | 8 | 13 | 1 |
| 2 | 0 | 8 | 7 | 1 |
| 4 | 2 | 7 | 14 | 1 |
| 6 | 4 | 10 | 24 | 1 |
| 2 | 0 | 9 | 4 | 1 |
| 2 | 2 | 6 | 7 | 1 |
| 4 | 0 | 6 | 11 | 1 |
| 9 | 4 | 6 | 24 | 0 |
| 7 | 3 | 8 | 16 | 0 |
